# Repellent effects of insecticides against *Aedes aegypti*: a systematic review

**DOI:** 10.1186/s13071-025-07140-z

**Published:** 2025-12-16

**Authors:** Leonardo Barbosa Koerich, Artur Metzker Serravite, Pedro Henryque de Castro, Julia Paula Rabelo, Pedro Horta Andrade, Daniel Milagre Marques, Marcos Horácio Pereira, Mauricio Roberto Vianna Sant’Anna, Nelder Figueiredo Gontijo, Juliana Maria Trindade Bezerra, Grasielle Caldas D.’Ávila Pessoa

**Affiliations:** 1 Laboratório de Entomologia Médica, Departamento de Parasitologia, Universidade Federal de Minas Gerais, Belo Horizonte, Minas Gerais Brazil; 2 Laboratório de Genômica Evolutiva, Departamento de Genética, Universidade Federal Do Rio de Janeiro, Rio de Janeiro, Brazil; 3 Laboratório de Fisiologia de Insetos Hematófagos, Departamento de Parasitologia, Universidade Federal de Minas Gerais, Belo Horizonte, Minas Gerais Brazil; 4 Centro de Estudos Superiores de Lago da Pedra, Universidade Estadual Do Maranhão, São Luís, Maranhão Brazil

**Keywords:** *Aedes aegypti*, Insecticide, Repellency, Resistance, HITSS, Excito-repellency, Vector control

## Abstract

**Background:**

*Aedes aegypti* is the primary vector of arboviruses, including dengue, Zika and chikungunya, representing a major global public health concern. Owing to the lack of effective vaccines and specific therapeutic options for these infections, vector control remains the main strategy to limit their spread. Traditionally, vector control has relied on extensive use of insecticides combined with the elimination of breeding sites. However, in addition to selecting for insecticide-resistant mosquitoes, concerns have arisen about behavioural effects induced by insecticides, particularly repellency – defined as the ability of a chemical compound to trigger avoidance behaviour in insects, thereby reducing their exposure to treated surfaces. This systematic review aimed to synthesise current knowledge on repellent effects of certain insecticides on *A. aegypti*.

**Methods:**

A literature search was conducted in the databases Virtual Health Library (BVS), PubMed® and Scientific Electronic Library Online (SciELO), following the Preferred Reporting Items for Systematic Reviews and Meta-Analyses (PRISMA) guidelines. A total of 46 original studies published between 1990 and 2023 were included.

**Results:**

Altogether, 433 bioassays were analysed, of which 69.8% reported repellent effects. The most common methods used to assess repellency were excito-repellency chambers, HITSS assays and the arm-in-cage test. Pyrethroids were used in 86.6% of repellency assays, followed by organochlorines (9.4%). Regarding the resistance profile of tested mosquito populations, susceptible populations exhibited higher frequencies of contact (92.2%) and spatial (77.3%) repellency behaviours than resistant ones (74.1% and 44.0%, respectively).

**Conclusions:**

Our findings indicate that insecticide-induced repellency is common and may interfere with the effectiveness of chemical control strategies. Nevertheless, studies addressing the underlying molecular and sensory mechanisms involved in repellent perception remain scarce.

**Graphical Abstract:**

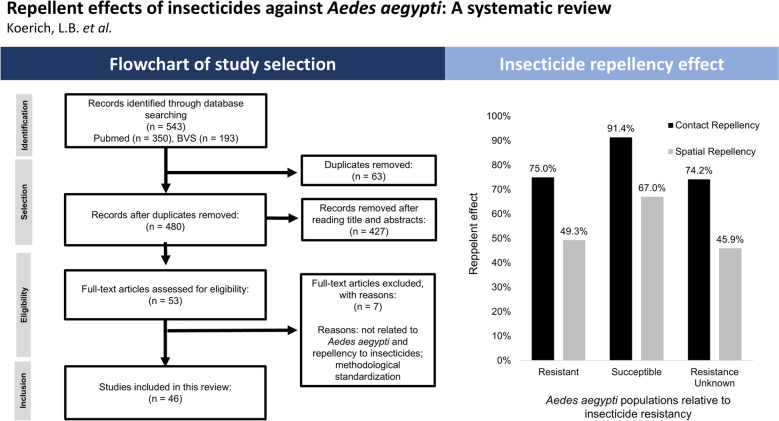

**Supplementary Information:**

The online version contains supplementary material available at 10.1186/s13071-025-07140-z.

## Background

*Aedes aegypti* is one of the most important arbovirus vectors worldwide, transmitting diseases such as dengue, Zika and chikungunya. According to the World Health Organization (WHO), more than six million people are infected annually by dengue viruses in over 100 countries [1]. The control of *A. aegypti* is one of the most effective strategies to reduce the transmission of these arboviruses, relying primarily on insecticides and the elimination of breeding sites, in conjunction with public health education campaigns [2]. However, insecticides can elicit behavioural effects in mosquitoes, including repellency. Compounds such as DDT and pyrethroids, beyond their insecticidal action, have demonstrated irritant and repellent properties [3, 4]. In this context, repellency is defined as the capacity of an insecticide to deter mosquitoes either before or after contact with a treated surface [5]. This avoidance behaviour can hinder the success of control interventions and potentially favour the selection of more tolerant populations.

Since the 1940s, studies have shown that exposure to insecticides can induce behavioural responses that reduce the duration of mosquito contact with treated surfaces [6–8]. The repellent effect can be categorised into two main types: spatial and contact repellency. Spatial repellency occurs when volatile compounds repel mosquitoes without direct contact, as observed with vapour-phase devices or diffusers [9]. Contact repellency, on the other hand, requires physical contact with a treated surface, triggering irritability and consequent escape behaviour [10, 11]. In both cases, the effects are mediated by chemosensory receptors located on the antennae and tarsi of mosquitoes [9].

The perception of repellent compounds occurs mainly through the activation or inhibition of olfactory receptors. For instance, the repellent DEET (*N,N*-diethyl-*m*-toluamide) acts on the insect nervous system, impairing its ability to locate and approach hosts [10]. Insecticide resistance is another factor associated with repellent behaviour. Knockdown resistance (*kdr*) mutations in the voltage-gated sodium channel gene have been linked not only to pyrethroid tolerance but also to heightened repellent responses in mosquitoes [12]. Likewise, metabolic resistance mechanisms, such as overexpression of cytochrome P450 genes (e.g. *CYP9J27*, *CYP6CB1*, *CYP9J26*, *CYP9M4*), may influence both perception and behavioural responses to insecticides [13–15].

Various methodologies have been developed to evaluate insecticide repellency in mosquitoes. One of the earliest standardised methods is the arm-in-cage test [16, 17], with established protocols by the WHO [18] and the US Environmental Protection Agency (EPA) [19]. This method assesses repellent efficacy by measuring landing or biting behaviour on treated versus untreated human skin. Another widely used technique is the excito-repellency chamber (ERC) assay, proposed by Roberts et al. [20], which evaluates mosquito escape from a chamber treated with insecticide. The ERC test allows differentiation between spatial and contact repellency effects, making it a versatile approach. A variant of the ERC method is the High Throughput Screening System (HITSS), developed by Grieco et al. [21], which employs a cylindrical tube divided into treated and untreated zones, enabling observation of mosquito behaviour upon exposure. The HITSS has gained popularity in the past decade due to its affordability and ease of use, allowing a large number of assays to be conducted within a short time frame. While the arm-in-cage test always involves an attractant, ERC and HITSS assays can be conducted with or without an attractant, which may influence the observed outcomes. Assays using attractants assess how the repellent interferes with the mosquito’s ability to detect and approach the host. By contrast, assays without attractants evaluate insect movement in response to a treated surface. These tests typically employ adult, non-blood-fed female mosquitoes owing to their heightened host-seeking behaviour, which enhances the sensitivity of repellency measurements [22–24].

Methodological choices, such as the repellent dose tested, the number of mosquitoes used, exposure duration and origin of the mosquito populations, also influence outcomes and must be carefully considered. Available protocols [18–21] provide general guidelines, focusing on physiological state (non-blood-fed nulliparous females), sample size and precautions when human volunteers are used (e.g. skin cleansing, avoiding scented products). Other decisions, such as dosing strategies, compound delivery methods and exposure times, are typically left to the researcher, resulting in considerable methodological heterogeneity that hinders comparative analysis across published studies.

In 2017, the WHO launched the Global Vector Control Response plan, with goals to reduce mortality from vector-borne diseases by up to 75% by 2030 [2]. Among the proposed strategies is the development of new insecticides and the use of spatial and topical repellents. In this context, to better understand the repellent effects of insecticides, we conducted a systematic review guided by the question: “What is currently known about insecticide-induced repellency in *Aedes aegypti*?” The objectives of this review were to: (i) compile evidence regarding whether insecticides cause repellency in *A. aegypti*, (ii) identify the most commonly used methodologies for repellency testing and their variants, (iii) determine whether the reviewed studies discussed behavioural mechanisms underlying repellency and (iv) investigate whether insecticide resistance influences the evasive behaviour of mosquitoes in response to products used in vector control.

## Methods

### Study design

The primary aim of this study was to compile information regarding the repellency of *Aedes aegypti* to insecticides, with a focus on the methodologies employed, the compounds tested, the mosquito populations used and the outcomes observed. This systematic review was guided by the question: “What is known about the repellent effects of insecticides on *A. aegypti*?” The review followed the Preferred Reporting Items for Systematic Reviews and Meta-Analyses (PRISMA) guidelines [25], and a PRISMA checklist is provided as Additional File 1.

### Search strategy

The literature search was conducted in January 2024, with a date range of 1990 to 2023. Controlled vocabulary descriptors from both the Health Sciences Descriptors (DeCS) and the Medical Subject Headings (MeSH) were used in Portuguese, English and Spanish (therefore, restricting our search to articles published in such languages). Boolean operators “AND” and “OR” were employed to enhance the search scope. The search terms included: (1) repellency, (2) insect repellents, (3) *Aedes aegypti*, (4) organochlorines, (5) pyrethroids, (6) organophosphates, (7) carbamates, (8) neonicotinoids and (9) insect growth regulators. The string used for the search is detailed in the Additional File 1. Besides the date range, no other filter was used.

### Databases consulted

The bibliographic search was conducted using the following electronic databases: Biblioteca Virtual de Saúde (BVS), Medline (via PubMed®) and Scientific Electronic Library Online (SciELO).

### Eligibility and inclusion criteria

Abstracts, theses, books or book chapters and review articles were excluded at the initial screening stage. Following the database searches, duplicate records were removed. Titles and abstracts of the remaining studies were then screened, and those outside the scope of the review were excluded. Full-text reading was conducted for the remaining articles. Studies that assessed the association and/or correlation of specific factors with the repellency of *Aedes aegypti* to insecticides were deemed eligible for systematic review. We also used the variables extracted (see Data Extraction from Selected Articles) to evaluate study quality, including only studies that contained information for at least 30 variables in this review. Two independent reviewers conducted all article inclusion and exclusion procedures; in cases of disagreement, a third reviewer was consulted to reach a final decision.

### Data extraction from selected articles

From the selected studies, 50 variables were extracted, focusing on: title, authors, publication year, *A. aegypti* populations studied, insecticide resistance/susceptibility profiles, physiological characteristics of mosquitoes used in the assays, methodology applied, insecticides tested, methodological details (e.g. sample size, assay timing, doses tested, use of positive controls, treated materials, attractants used, pre-assay and intra-assay care, etc.), outcomes observed and statistical analyses performed. The complete list of extracted data and categorisation is available in Additional File 2.

## Results

**Fig. 1:**
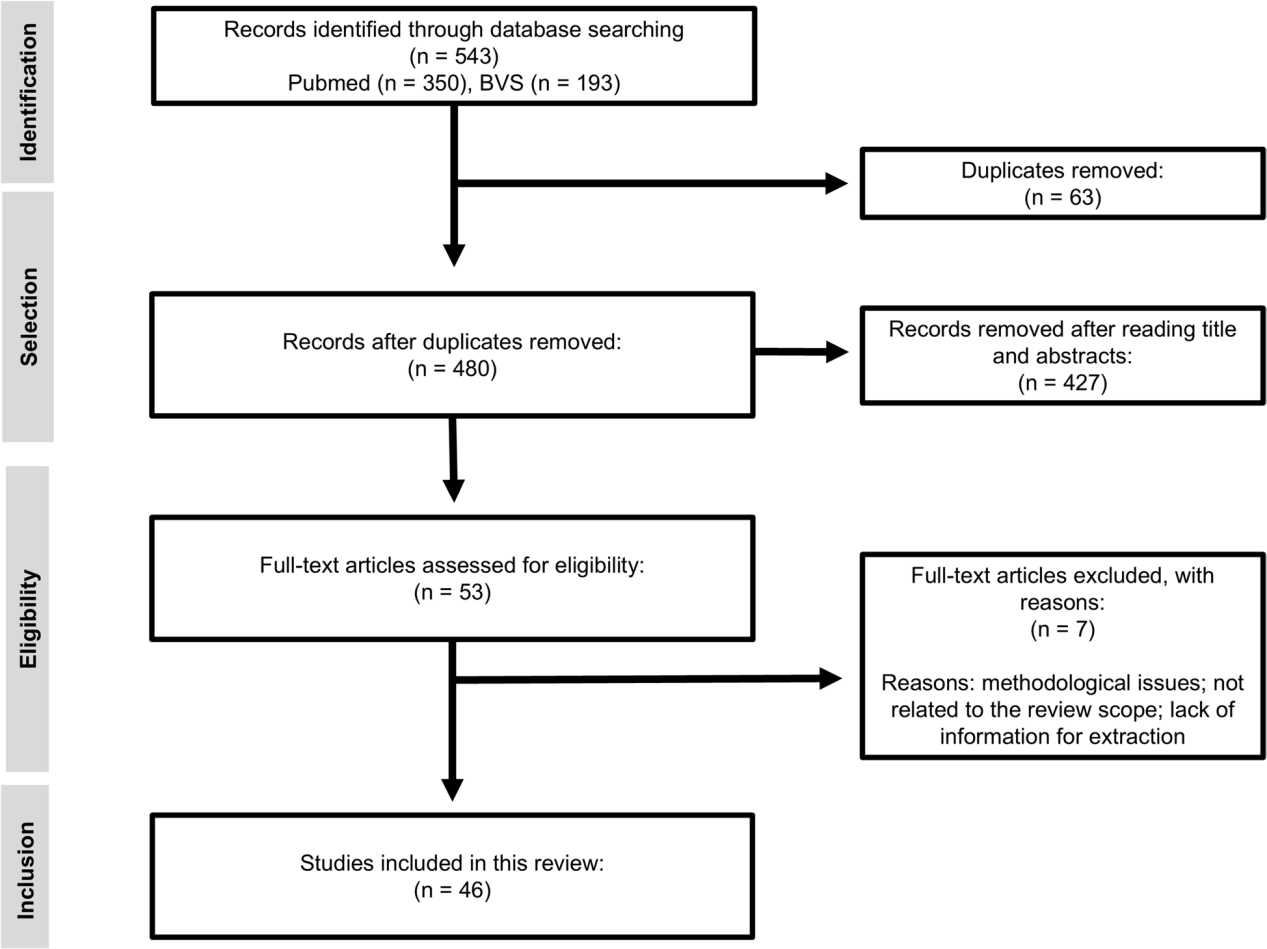
Flowchart of scientific article selection across electronic databases for the systematic review on insecticide repellency in *Aedes aegypti*

A total of 543 studies were initially identified through the bibliographic search. Of these, 63 were excluded due to duplication, and a further 427 were excluded after screening titles and abstracts, resulting in 53 studies selected for full-text review. After this stage, seven studies were excluded for not conducting repellency assays with insecticides on *A. aegypti*, or for being focused solely on methodological standardisation. Ultimately, 46 studies were included in this systematic review (Fig. 1). An increasing trend in the number of studies on *A. aegypti* repellency to insecticides has been observed over the years, with more than 60% of the articles published in the last 15 years. Across the 46 studies, a total of 751 repellency bioassays were identified. However, 18 studies tested varying doses to assess dose–response relationships (Table 1). When a study evaluated multiple insecticide dosages, we chose to include only the results from the lowest effective dose, which provides a more conservative and representative picture of the repellency effect, ensuring that high-dose experiments do not skew conclusions. Thus, 433 assays were considered in the final analysis for this review. We considered an effect to be repellent if the study reported statistically significant behavioural metrics, including changes in escape percentage, landing rates, probing activity, or feeding rates.

**Table 1:** Selected studies on insecticide repellency in *Aedes aegypti*: summary of methodology and bioassay outcomes

References	Method used	Insecticide tested (compound)	Contact repellent effect			Spatial tepellent effect		
			Resistant insects	Susceptible insects	Unknown resistance	Resistant insects	Susceptible insects	Unknown resistance
Andreazza et al. [31]	HC	Transflurithrin (Pyr)				Yes* (1L)	Yes* (2L)	
Sougoufara et al. [30]	FE	Proxopur (Car)		Yes (1L)			Yes (1L)	
	FE	Malathion (Oph)		Yes (1L)			Yes (1L)	
	FE	Deltamethrin (Pyr)		Yes (1L)			Yes (1L)	
	FE	λ-cyhalothrin (Pyr)		Yes (1L)			Yes (1L)	
	FE	Permethrin (Pyr)		Yes (1L)			Yes (1L)	
Valbon et al. [32]	HC	Bioalletrhin (Pyr)					Yes* (4L)	
Andreazza et al. [33]	HC	Transflurithrin (Pyr)				Yes* (1L, 1FP)	Yes* (3L)	
Liu et al. [34]	HC	Pyrethrum (Pyr)				Yes*(1L)	Yes*(4L)	
Yu et al. [70]	ERC	Deltamethrin (Pyr)		Yes (1L)	No (10FP)			
	ERC	Deltamethrin (Pyr) + adjuvant		Yes (1L)	Yes (10FP)			
Yu et al. [71]	ERC	Deltamethrin (Pyr)	Yes (8FP)	No (1L) / Yes (8FP)		Yes (8FP)	No (1L) / Yes (8FP)	
	ERC	Permethrin (Pyr)	Yes (8 FP)	No (1L) / Yes (8FP)		Yes (8 FP)	No (1L) / Yes (8FP)	
Dhiman et al. [42]	ERC	Deltamethrin (Pyr)			Yes (1FP)			Yes (1FP)
Sukkanon et al. [45]	ERC	Transflurithrin		Yes* (1L)	Yes* (1FP)		Yes* (1L)	Yes* (1FP)
Estep et al. [72]	HC	Permethrin (Pyr)	No (1FP)	Yes (1L)				
Paiz-Moscoso et al. [73]	HITSS	Transflurithrin (Pyr)			Yes* (3L)			Yes* (4L)
Sathantriphop et al. [74]	ERC	Cypermethrin (Pyr)	Yes* (2FP)	Yes* (1L)		No* (2FP)	No* (1L)	
	ERC	Deltamethrin (Pyr)	Yes* (2FP)	Yes* (1L)		No* (2FP)	No* (1L)	
Yang et al. [59]	Spatial Essay	Metofluthrin (Pyr)					Yes* (1L, 2FP)	
	Spatial Essay	Permethrin (Pyr)					Yes* (1L, 2FP)	
	Spatial Essay	Transflurithrin (Pyr)					Yes* (1L, 2FP)	
Estrada et al. [75]	HITSS	Transflurithrin (Pyr)			Yes (1L)			
Jiang et al. [63]	HITSS	Permethrin(Pyr)						No* (1L)
	HITSS	Transflurithrin (Pyr)						Yes* (1L)
Bowman et al. [12]	HC	Deltamethrin (Pyr)		Yes (4FP)			Yes (4FP)	
	HC	Etofenprox (Pyr)		Yes (4FP)			Yes (4FP)	
	HC	Permethrin (Pyr)		Yes (4FP)			Yes (4FP)	
McPhatter et al. [44]	FE	Transflurithrin (Pyr)			Yes* (1L)			Yes* (1L)
Agramonte et al. [76]	HC	DDT (Org)	Yes* (1FP)	Yes* (1L)				
	HC	Deltamethrin (Pyr)	Yes* (1FP)	Yes* (1L)				
	HC	Etofenprox (Pyr)	Yes* (1FP)	Yes* (1L)				
	HC	Permethrin (Pyr)	Yes* (1FP)	Yes* (1L)				
Nentwig et al. [77]	Y-olfact	Transflurithrin (Pyr)					Yes* (1L)	
	Special Cage	Transflurithrin (Pyr)					Yes* (1L)	
	House Wall	Transflurithrin (Pyr)					Yes* (1L)	
Orsborne et al. [29]	HC	Permethrin (Pyr)		Yes* (1L)		Yes* (1L)		
	House Wall	Permethrin (Pyr)			No* (1FP)			Yes* (1FP)
Ponlawat et al. [43]	FE	Metofluthrin (Pyr)						No(1L)
Wagman et al. [78]	HITSS	Transflurithrin (Pyr)					No (3 FP) / Yes (2L; 3FP)	
DeRaedt Banks et al. [51]	HC	Permethrin (Pyr)		Yes (3L)				
Sukumaran et al. [27]	HC	Permethrin (Pyr)						Yes (1ND)
Sathantriphop et al. [79]	ERC	Deltamethrin (Pyr)		Yes (1L)			Yes (1L)	
	ERC	Permethrin (Pyr)		Yes (1L)			Yes (1L)	
Canyon and Muller [28]	Oviposition	Malathion (Oph)						No (2ND)
		Temephos (Oph)						No (2ND)
		Permethrin (Pyr)						No (2ND)
Manda et al. [80]	FE	Alphacypermethrin (Pyr)		No* (2FP)				
	ERC	Alphacypermethrin (Pyr)		Yes* (1FP)				
	ERC	Deltamethrin (Pyr)		Yes* (1FP)				
	ERC	λ-Cyhalothrin (Pyr)		Yes* (1FP)				
Faulde and Nehring [81]	HC	Etophenprox (Pyr)			Yes* (1L)			Yes* (1L)
		Permethrin (Pyr)			No*(1L) / Yes*(1L)			No* (2L)
Boonyuan et al. [46]	ERC	Deltamethrin (Pyr)			Yes* (2FP)			No*(2FP)/ Yes* (2FP)
Pennetier et al. [26]	FE	Permethrin (Pyr)			Yes (4ND)			
Thanispong et al. [36]	HITSS	DDT (Org)	Yes* (6FP)			No*(3FP) / Yes* (3FP)		
	HITSS	Alphacypermethrin (Pyr)			Yes* (6 FP)			No* (6 FP)
	HITSS	Deltamethrin (Pyr)		Yes* (6FP)			No*(2FP) / Yes* (4FP)	
	HITSS	Permethrin (Pyr)		No*(1FP) / Yes* (5FP)			No*(5FP) / Yes* (1FP)	
Kramer et al. [82]	Other	Alphacypermethrin (Pyr)		No* (1L)				
Thanispong et al. [69]	ERC	Alphacypermethrin (Pyr)		Yes (1L, 2FP)			No (FP) / Yes (1L, 2FP)	
	ERC	DDT (Org)		Yes (1L, 2FP)			Yes (1L, 2FP)	
Cooperband and Allan [83]	Cage	Biphentothrin (Pyr)			Yes (1ND)			
	Cage	Deltamethrin (Pyr)			Yes (1ND)			
	Cage	λ-cyhalothrin (Pyr)			Yes (1ND)			
Achee et al. [9]	HITSS	Bendiocarb (Car)	Yes* (1FP)			No* (1FP)		
	HITSS	Proxopur (Car)	Yes* (1FP)			No* (1FP)		
	HITSS	Chlorodane (Org)	No* (1FP)			No* (1FP)		
	HITSS	DDT (Org)	Yes* (1FP)			No* (1FP)		
	HITSS	Dieldrin (Org)	No* (1FP)			No* (1FP)		
	HITSS	Methoxychlor (Org)	Yes* (1FP)			No* (1FP)		
	HITSS	Fenitrothion (Oph)	Yes* (1FP)			No* (1FP)		
	HITSS	Malathion (Oph)	Yes* (1FP)			No* (1FP)		
	HITSS	Alphacypermethrin (Pyr)	Yes* (1FP)			No* (1FP)		
	HITSS	Cypermethrin (Pyr)	Yes* (1FP)			No* (1FP)		
	HITSS	Deltamethrin (Pyr)	Yes* (1FP)			No* (1FP)		
	HITSS	Permethrin (Pyr)	Yes* (1FP)			No* (1FP)		
Mongkalangoon et al. [47]	ERC	Cyphenotrin (Pyr)			Yes* (1FP)			Yes* (1FP)
	ERC	Deltamethrin (Pyr)			Yes* (1FP)			Yes* (1FP)
	ERC	Tetramethrin (Pyr)			Yes* (2FP)			Yes* (2FP)
Said et al. [40]	HITSS	DDT (Org)			Yes* (1FP)			Yes* (1FP)
	HITSS	Alphacypermethrin (Pyr)			Yes* (1FP)			No* (1FP)
	HITSS	Deltamethrin (Pyr)			Yes* (1FP)			No* (1FP)
	HITSS	Permethrin (Pyr)			Yes* (1FP)			No* (1FP)
Polsomboon et al. [64]	ERC	DDT (Org)	Yes (4FP)			Yes (4FP)		
	ERC	Deltamethrin (Pyr)		Yes (4FP)			Yes (4FP)	
Ayala-Sulca et al. [84]	HITSS	DDT (Org)			Yes* (1FP)			No* (1FP)
		Biphentrin (Pyr)			Yes* (1FP)			No* (1FP)
		Permethrin (Pyr)			Yes* (1FP)			No* (1FP)
Kawada et al. [37]	FE	Metofluthrin (Pyr)			Yes (1FP)			
Paeporn et al. [38]	ERC	Deltamethrin (Pyr)	NSA (1FP)	NSA (1L)		NSA (1FP)	NSA (1L)	
	ERC	Permethrin (Pyr)	NSA (1FP)			NSA (1FP)		
Chareonviriyaphap et al. [85]	ERC	Cypermethrin (Pyr)	No (9FP) / Yes (3FP)			No (10FP) / Yes (2FP)		
	ERC	Deltamethrin (Pyr)	No (9FP) / Yes (3FP)			No (8FP) / Yes (4FP)		
Kongmee et al. [86]	ERC	Deltamethrin (Pyr)	Yes (6FP)	Yes (3L)		No (6FP)	No (3L)	
Rutledge et al. [41]	Other	Permethrin (Pyr)			Yes* (2 L)			
Karch et al. [39]	FE	Deltamethrin (Pyr)			No (1FP)			
Schreck, [35]	FE	Permethrin (Pyr)		Yes (1L)				

*HC* hand in cage, *ERC* excito-repellency chamber, *FE* field experiment, *Car* Carbamate, *Pyr* pyrethroid, *Oph* organophosphate, *Org* organochlorate, the parentesis after repellency effect shows the number of assays with laboratory populations (L), field populations (FP) or populations with unknown origin (ND) with the same repellency result; * assays that tested variable dosages and had same result; *NSA* no statistical analysis

### Populations of *Aedes aegypti* used in the studies

Across the 46 selected studies, 47 field populations and 22 laboratory populations of *A. aegypti* were tested, including two genetically modified lines targeting the Orco and AaOr31 olfactory receptors. Of the 433 bioassays analysed, 322 (74.4%) used field populations, 95 (22.0%) used laboratory populations (including five with genetically modified mosquitoes) and 16 assays (3.7%) from four studies did not specify the population origin [26–30]. Among the laboratory strains, most assays employed mosquitoes reared for multiple generations within the institution (34 assays; 7.9%). The Rockefeller strain, widely regarded as a reference for toxicological studies, was used in only four assays (0.9%), and the Liverpool genomic reference strain in two assays (0.5%). The resistant reference strain Rockefeller^*kdr*^ was tested in four assays. Four studies used genetically modified lines [31–34] targeting olfactory receptors, namely Orco^−/−^ (3 assays) and AaOr31^−/−^ (2 assays).

A total of 38 mosquito populations were evaluated for insecticide susceptibility. Of these, 34 assessments (89.5%) employed the standard WHO diagnostic test, five of which also investigated *kdr* mutations and esterase overexpression (including the Rockefeller^*kdr*^ strain). The remaining four populations were assessed using the CDC diagnostic test, with three also mapping *kdr* mutations. Of the 433 assays, 165 (38.1%) involved resistant populations, 169 (39.0%) susceptible populations and 99 (22.9%) populations with undetermined resistance profiles.

### Methodologies used in the assays

Most of the studies were conducted in laboratory settings (40 out of 46), accounting for 97.5% of the assays (422). Regarding the methods used to evaluate repellency (Table 1), 13 studies (28.3%) employed excito-repellency chambers; 12 (26.1%) used the hand-in-cage method; eight (17.4%) adopted the HITSS system; and seven (15.2%) conducted experiments in field or semi-field conditions. The remaining six studies (13.0%) used other methods, such as the WHO tunnel test (commonly used to measure excito-repellency of impregnated surfaces), Y-tube olfactometers, specially designed cages, spatial assays, treated mesh, or custom devices developed for the specific study.

When evaluating the total number of assays per methodology (Additional file 2), 233 (53.8%) used excito-repellency chambers, 102 (23.6%) used HITSS, 51 (11.8%) employed the hand-in-cage test, 10 assays (2.3%) used the WHO tunnel test, 7 (1.6%) used spatial assays, 4 (0.9%) employed Y-tube olfactometers or special cages and 3 (0.7%) assessed oviposition repellency. Field assays focused on testing treated walls (10 assays; 2.3%) or impregnated tents (3 assays; 0.7%). A total of six assays (1.4%) used custom methodologies designed by the researchers. Roughly half of the assays (212) tested for spatial repellency, while the other half (221) assessed contact repellency (Additional File 2).

Regarding the mosquitoes used, the majority of assays (328; 75.7%) employed 2–10 day-old females fed exclusively on sugar and subjected to 24-h fasting before experiments (Table 2). Conversely, 62 assays (14.3%) across six studies [26, 35–39] did not specify the physiological status of the mosquitoes. Two studies employed male mosquitoes [40, 41]. In 357 assays (82.5%), the sample size ranged from 10 to 30 insects per replicate, with only 12 assays using more than 100 mosquitoes. Over 70% of assays (322; 74.4%) performed acclimatisation before testing. Of the assays, 276 (63.8%) were conducted during the daytime, while 147 (33.9%) did not specify the time, and only 10 (2.3%) were conducted at night. Exposure times to insecticides varied from 5 min to over 1 h. Most assays (226; 52.2%) used exposure periods between 21 and 30 min. Two studies [26, 30] tested exposure periods exceeding 60 min (17 assays; 3.9%).

**Table 2:** Data on sample number, insect physiology, acclimatisation, time of exposure to insecticides, use of attractants and positive control

Data on insects	Assays	%	Data on assays	Assays	%
Populations used			Time of exposure		
Field	322	74.4%	< 5 min	7	1.6%
Laboratory	90	20.8%	5 to 20 min	159	36.8%
Genetically modified	5	1.2%	21 to 30 min	226	52.2%
Not specified	16	3.7%	31 to 60 min	17	3.9%
			> 61 min	17	3.9%
			Not specified	7	1.6%
Insect physiology			Attractants		
2–10 day sugar fed females	376	88.1%	Human	66	15.2%
2–10 day blood fed females	20	4.7%	Mice	14	3.2%
Gravid females	8	1.9%	Blood	2	0.5%
Parous females	4	0.9%	CO^2^	1	0.2%
5–10 day males	9	2.1%	No attractants used	350	80.8%
Not specified	14	3.3%			
			Positive control		
Number of insects used			DEET	22	5.1%
< 10	1	0.2%	DEET + Linalol	7	1.6%
10 to 30	357	82.5%	DEET + Undecanone + IR3535	6	1.4%
31 to 60	33	7.6%	DEET + Picardin	4	0.9%
61 to 100	25	5.8%	Picardin	4	0.9%
> 100	12	2.8%	Acetate Geranil	3	0.7%
Not specified	5	1.2%	DEET + IR3535	2	0.5%
			No positive control	385	88.9%
Insect acclimatization					
5–30 min	322	74.4%	The time the assay was conducted		
No climatization	111	25.6%	Day	276	63.8%
			Night	10	2.3%
			Not specified	147	33.9%

*DEET* N,N-diethyl-3-methylbenzamide; *IR3535* ethyl butylacetylaminopropionate

Of the 433 assays, 350 (80.8%) were conducted without any attractant (Table 2). When used, human attractants were most common (66 assays; 15.2%), followed by mice or rats (14; 3.2%), blood (2; 0.5%) and CO_2_ (1; 0.2%). Similarly, 385 assays (88.9%) did not include a positive control. When applied, DEET was the most common positive control, either alone (22 assays; 5.1%) or in combination with other substances such as linalool (7; 1.6%), picaridin (4; 0.9%), IR3535 (2; 0.5%) or DEET + IR3535 + undecanone (6; 1.4%).

Among the 46 studies, 27 (58.7%) did not mention any specific pre-assay or intra-assay precautions. The most frequent precaution among those that did was the impregnation of materials up to 24 h before the assays (8 studies; 17.3%). Of the 12 hand-in-cage studies, only five described precautions such as washing the skin with soap and/or ethanol and avoiding the use of lotions or perfumes before testing. No studies reported details about the human volunteers (e.g. sex, age, alcohol or drug consumption). Other reported precautions included pre-exposure of mosquitoes to insecticides or solvents (one study; 2.2%), a 90-min interval between trials (one study; 2.2%), behavioural testing in untreated chambers (one study; 2.2%), washing treated materials (one study; 2.2%) and use of heavily worn impregnated materials (two studies; 4.4%).

Concerning the insecticides tested (Table 3), pyrethroids were the most frequently evaluated chemical class, with their repellency assessed in 377 assays (88.3%), followed by organochlorines (40 assays; 9.4%), organophosphates (10 assays; 2.8%) and carbamates (6 assays; 1.4%). Deltamethrin and permethrin were the most studied insecticides, used in 155 (36.3%) and 92 (21.5%) of the assays, respectively. Other pyrethroids frequently evaluated included transfluthrin (36 assays; 8.3%), cypermethrin (32; 7.4%) and alpha-cypermethrin (26; 6.0%). Among the organochlorines, DDT was employed in 34 assays (8.0%), whereas chlordane, dieldrin and methoxychlor were each tested in two assays (0.5%). Of the organophosphates, malathion was the most used (six assays; 1.4%), while fenitrothion and temephos were each assessed in two assays (0.5%). Lastly, two carbamates were used in repellency assays: propoxur (4 assays; 0.9%) and bendiocarb (2 assays; 0.5%). Technical-grade insecticides were used in 416 assays (96.1%). Seven studies [26, 34, 35, 37, 39, 42–44] tested formulated insecticides, totalling 12 assays (2.8%). The study by Liu et al. [34] did not specify the source or grade of the insecticide used in its five assays (1.2%). More than half of the assays used insecticides diluted in acetone (172 assays; 39.7%) or acetone combined with oil (144; 33.3%) (Additional file 2). Other reported diluents included ethanol (12 assays; 2.8%), oil alone (10; 2.3%), unspecified resin (3; 0.7%) and alkyd resin (2; 0.5%). Sixteen studies did not report how insecticides were diluted, accounting for 90 assays (20.8%). Regarding the concentrations tested for repellency, only four studies [30, 45–47] stated that the doses were based on lethal dose values (ranging from LD_30_ to LD_90_) (Additional file 2). The remaining studies did not provide any rationale for the dose selection.

**Table 3:** Number of assays for each insecticide tested for repellency against *A. aegypti*

Insecticide tested	Assays	%
Pyrethroid	**377**	**88.3%**
Deltamethrin	155	36.3%
Permethrin	92	21.5%
Transfluthrin	36	8.3%
Cypermethrin	32	7.4%
α-Cypermethrin	26	6.0%
Pyrethrum	7	1.6%
Bioallethrin	4	0.9%
λ-Cyhalothrin	4	0.9%
Metofluthrin	4	0.9%
Tetramethrin	4	0.9%
Bifenthrin	3	0.7%
Cyphenothrin	2	0.5%
Etofenprox	4	0.9%
Organochlorate	**40**	**9.4%**
DDT	34	8.0%
Chlorodane	2	0.5%
Dieldrin	2	0.5%
Methoxychlor	2	0.5%
Organophosphate	**10**	**2.3%**
Malathion	6	1.4%
Fenitrothion	2	0.5%
Temephos	2	0.5%
Carbamate	**6**	**1.4%**
Propoxur	4	0.9%
Bendiocarb	2	0.5%

### Repellent effects of insecticides on *Aedes aegypti*

For this section, we did not include the results of six assays in Paeporn 2007 [38] owing to the lack of statistical analysis to determine the repellent effect, and therefore, the repellent effect was evaluated for a total of 427 assays (instead of 433). Among the 427 assays analysed, 299 (70.0%) reported repellent effects, while 128 (30.0%) did not observe any evidence of repellency in *A. aegypti* when exposed to insecticides (Additional file 2). In 63 of the 427 assays (14.8%), repellency was not assessed in isolation, but as part of other behavioural responses such as escape rate, flight initiation, interruption of feeding or changes in oviposition preference. In 270 assays (63.2%), the outcome was clearly described as ‘repellency’, with authors interpreting a reduction in the number of insects on or near the treated substrate, compared with control, as indicative of repellency. Of the 46 studies included in this review, 33 (71.7%) detected some level of repellency in at least one of their assays.

**Fig. 2:**
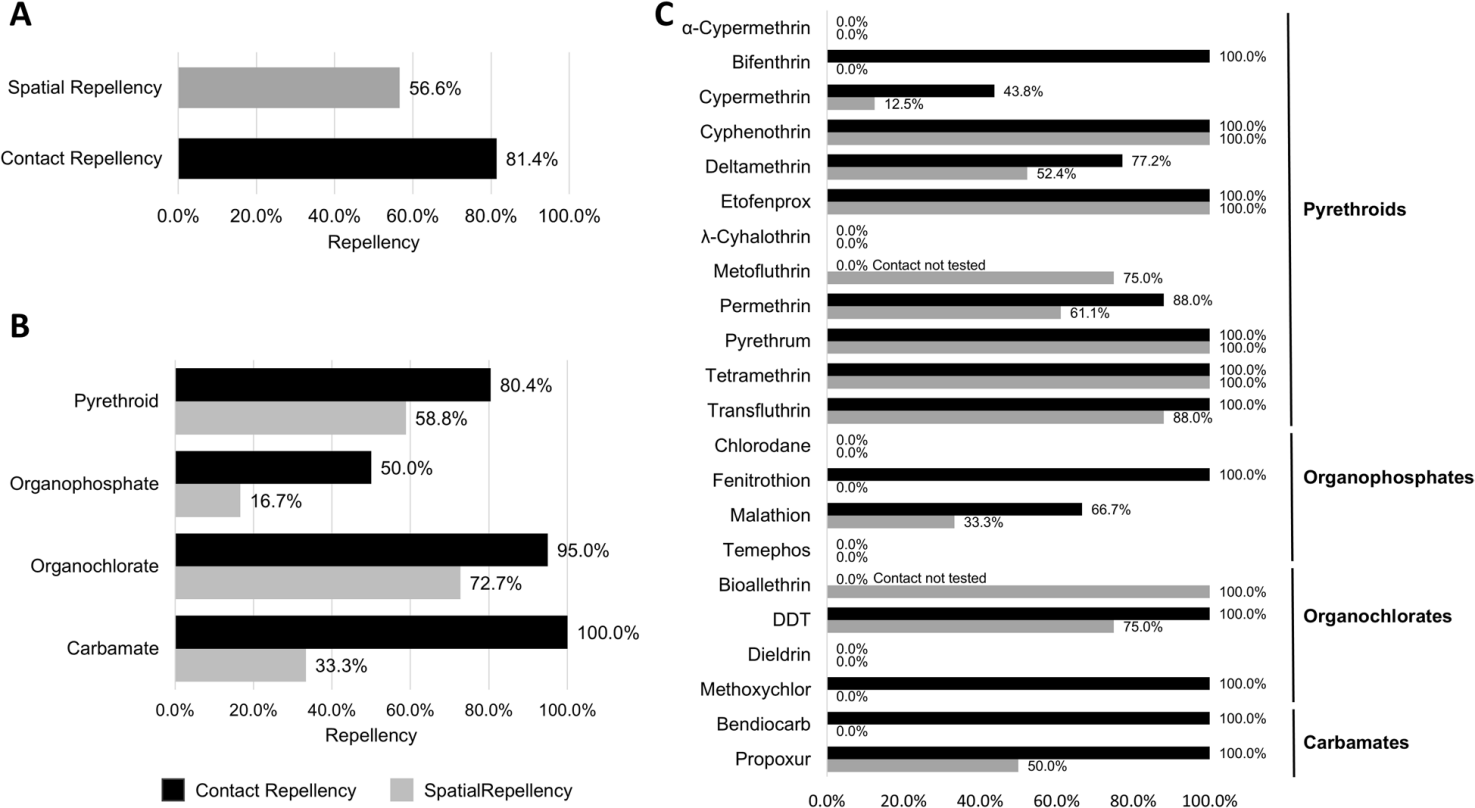
Frequency of contact and spatial repellency in resistant and susceptible *Aedes aegypti* populations. **A**. General contact and spatial repellency. **B**. Contact and spatial repellency by the four organic compounds. **C**. Contact and spatial repellency by the insecticides evaluated, grouped by organic compounds

Among the 181 assays conducted with susceptible mosquito populations, 92.2% of the assays that tested for contact repellency (85/93) reported some degree of repellency. In contrast, a repellency effect was observed for 67.0% of the assays that tested for spatial repellency (59/88). On the other hand, among the 147 assays conducted with resistant populations, contact repellency was observed in 74.1% of the contact assays (57/76) and only 44.0% for spatial repellency in spatial assays (35/71) (Fig. 2).

**Fig. 3:**
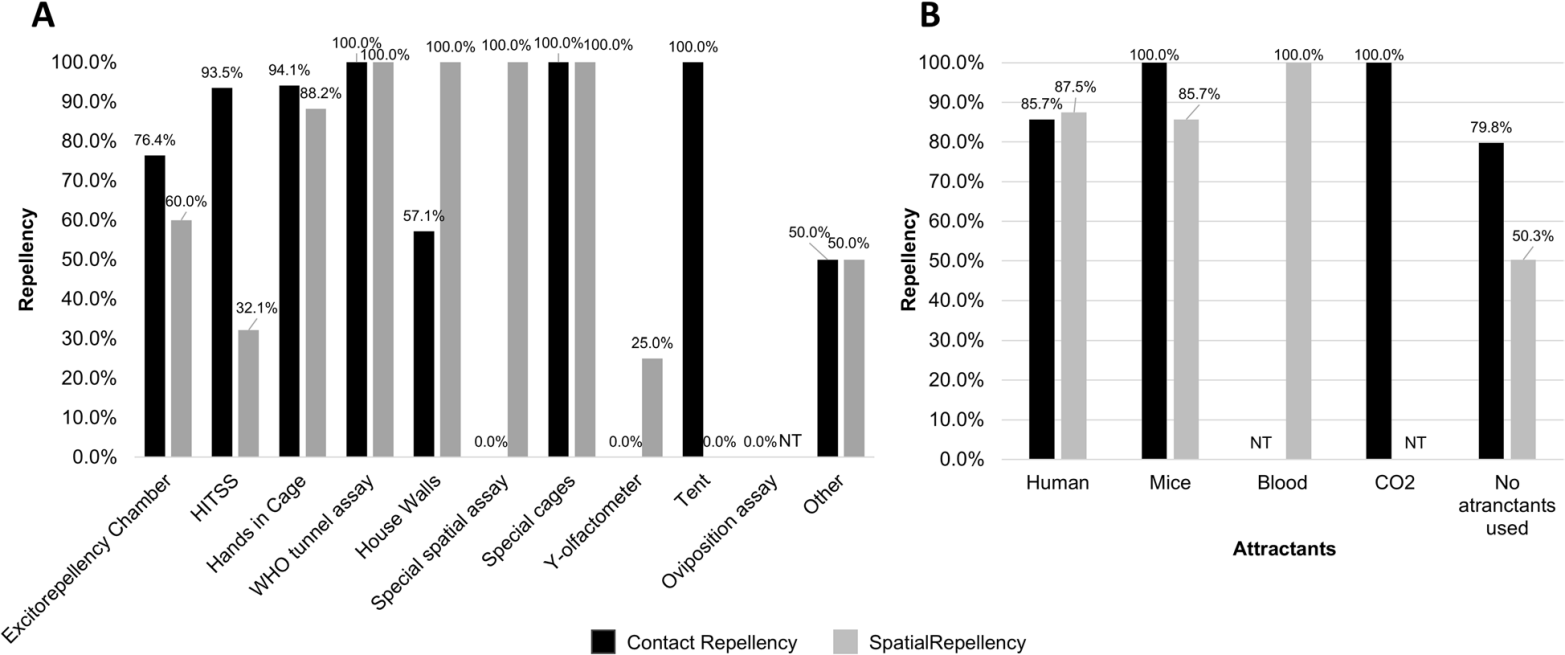
Contact and spatial repellency effects in *Aedes aegypti* according to methodological variables. **A** – Contact and spatial repellency observed in different types of assays; **B** – Contact and spatial repellency according to attractant type; *NT* Not tested

Regarding methodological aspects (Fig. 3), studies using excito-repellency chambers reported repellent effects in 76.4% (97/127) of contact repellency assays and 60.0% (60/100) of spatial repellency assays (Fig. 3A). Repellency assays using HITSS exhibited the greatest disparity between contact and spatial repellency, respectively 93.5% (43/46) and 32.5% (18/56). Among the three most commonly used methods, the hand-in-cage technique showed the highest proportion of repellent effects, with contact repellency observed in 94.1% (32/34) and spatial repellency in 88.2% (15/17) of assays. WHO tunnel tests and assays using specialised cages showed 100% repellent effects in both contact and spatial assays (5/5 each). By contrast, assays conducted on impregnated walls were the only ones in which contact repellency was reported less frequently, 57.1% (4/7), than spatial repellency, 100.0% (3/3). Contact repellency was not evaluated in assays using Y-tube olfactometers or spatial assays conducted in special chambers.

A majority of the assays were performed without any type of attractant (250 assays), and contact repellency was observed in 79.8% (146) of these, while spatial repellency occurred in 50.3% (84) (Fig. 3B). Humans were the most commonly used attractant (66 assays), with similar frequencies of contact (85.7%; 36/42) and spatial repellency (87.5%; 21/24). When mice were used as attractants, contact repellency was observed in 100% (7/7) of the assays, and spatial repellency in 85.7% (6/7). Most assays used female mosquitoes aged 2–10 days post-emergence, fed exclusively on sugar. In females aged 2–4 days post-emergence, contact repellency was observed in 69.2% (27/39) of assays, and spatial repellency in 62.8% (27/43) (Fig. 3C). For females aged 5–10 days post-emergence (fed only on sugar), contact repellency was reported in 84.4% (119/141) and spatial repellency in 65.7% (69/105) of assays. Contact and spatial repellency were observed in up to 50% of assays using blood-fed or gravid females (respectively, 9/9 and 6/6), and in 100% (4/4) of assays using females that had already oviposited.

Most assays (316) reported acclimating insects before exposure to insecticides. The results for contact and spatial repellency were similar between acclimated and non-acclimated mosquitoes (Fig. 3D), with 80.1% (133/166) contact repellency and 56.7% (85/150) spatial repellency for acclimated insects, and 82.9% (34/41) and 70.8% (17/24) for contact and spatial repellency, respectively, in non-acclimated insects.

**Fig. 4:**
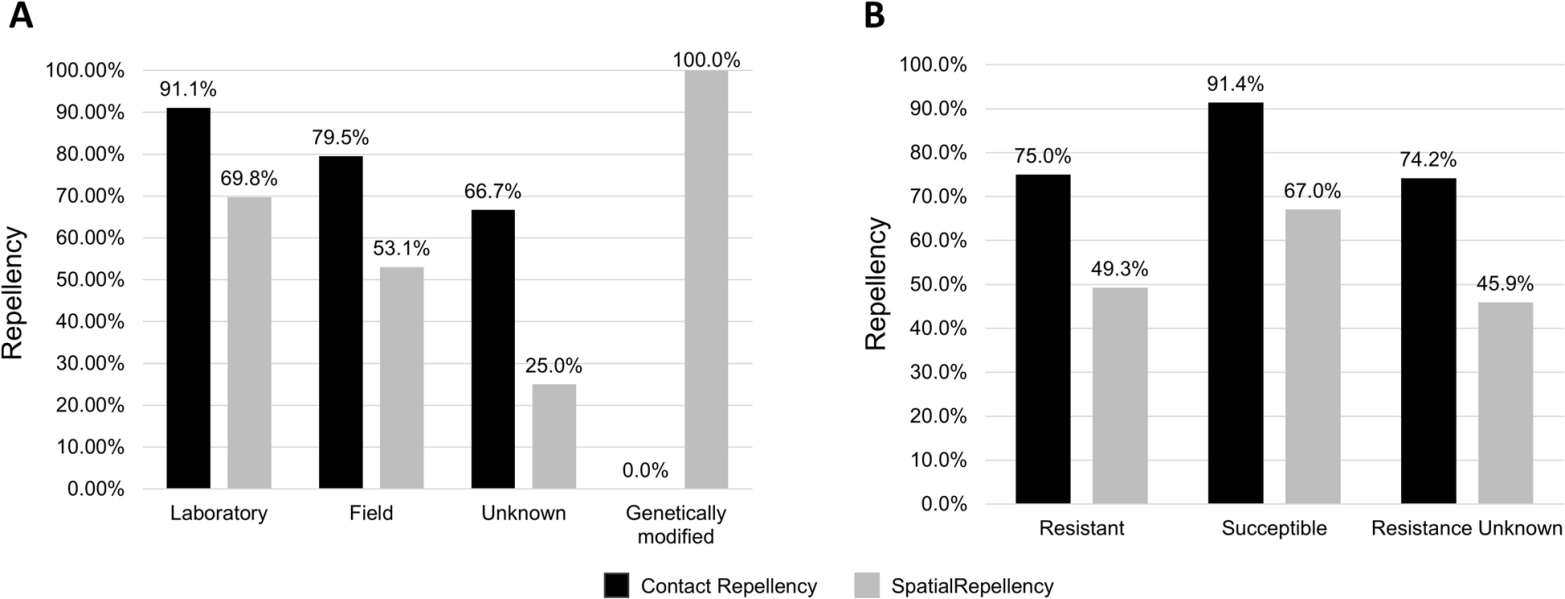
Contact and spatial repellency in *Aedes aegypti* according to population origin and insecticide susceptibility profile. **A** Contact and spatial repellency observed in laboratory, field, genetically modified and unspecified mosquito populations. **B** Contact and spatial repellency in insecticide-resistant and susceptible mosquitoes. Pearson’s chi-square test for independent grouped data was applied to assess the hypothesis that insecticide-resistant populations exhibit reduced evasive behaviour compared with susceptible mosquitoes (ns = not significant; *** = significance at *p* < 0.001). *NT* Not tested

Regarding the origin of mosquito populations and their insecticide resistance profiles (Fig. 4), repellent effects were observed in 91.1% (41/45) of contact repellency assays conducted with laboratory populations and 69.8% (30/43) of spatial repellency assays (Fig. 4A). For field populations, contact repellency was reported in 79.5% (136/171) and spatial repellency in 53.1% (78/147) of assays. Of the five assays conducted with genetically modified mosquitoes bearing knockouts of olfactory receptor genes, 100% demonstrated repellent effects (only spatial repellency was evaluated in these cases).

When repellency outcomes were assessed on the basis of the insecticide resistance status of the mosquitoes (Fig. 4B), contact repellency was observed in 75.0% (57/76) of assays with resistant mosquitoes and 91.4% (85/93) of assays with susceptible mosquitoes. When spatial repellency was evaluated, 49.3% (35/71) of the assays with resistant mosquitoes presented repellent behaviour, while 67.0% (59/88) of the susceptible mosquitoes presented the same effect. Mosquitoes whose susceptibility to the insecticide was not previously assessed showed levels of repellency similar to those of resistant insects, of 74.2% (46/62) for contact repellency and 45.9% (17/37) for spatial repellency.

## Discussion

Repellency is an avoidance behaviour mediated by sensory stimuli that prevent insects from coming into contact with a host or surfaces treated with organic or inorganic compounds. This phenomenon has been scientifically described since the earliest studies with volatile compounds such as DDT and permethrin, which demonstrated the potential of these chemicals to prevent mosquito landing and feeding even prior to the insect’s contact with the active substance [7, 8, 11]. The concept has evolved to distinguish between different forms of repellency: spatial repellency, which prevents the insect from approaching the stimulus source; and contact repellency, which occurs after physical contact with treated surfaces [10, 11, 48]. A detailed understanding of these effects is fundamental in the context of vector control, especially considering the increase in insecticide resistance, which threatens the effectiveness of tools such as insecticide-treated nets and residual spraying. This systematic review compiled studies on the repellent effect of insecticides on *Aedes aegypti*, identifying 46 scientific articles published between 1990 and 2023. It aimed to understand not only the evidence of repellent effects on susceptible and resistant insects to the tested insecticides, but also to survey the main methodological decisions in repellency assays and the potential impact of these decisions on result interpretation. However, the interpretation of our findings should be considered in light of potential publication bias, as the observed insecticide repellency effect may be influenced by the tendency for studies with positive results to be more frequently published.

Among standardised protocols for mosquito repellency testing, three stood out for having been widely used in the assessed studies: the hand-in-cage assay [18]; the excito-repellency chamber assay [49]; and the HITSS assay [21]. Each of these assays has different testing parameters, particularly regarding the sample size of insects (10–30 for HITSS; > 100 for hand-in-cage), the type of repellency assessed (spatial for HITSS; contact and/or spatial for hand-in-cage and excito-repellency chamber), the use of attractants (mandatory in the hand-in-cage test) and the criteria for determining repellency (evasion, landing and/or biting). In addition to the parameters defined for each assay, there are also those left to the researchers’ discretion, such as the chemicals tested, the tested doses and the impregnated materials (e.g. clothing, screens). Consequently, there is great methodological variability across the evaluated studies. This observed heterogeneity affects not only quantitative data but also scientific reproducibility, a point increasingly discussed in literature [22]. As an example, while the disparate designs of laboratory, semi-field and field studies introduce complexity in comparing results, our analysis found that only a small fraction of the assays (less than 3%) were conducted in semi-field or field settings. Since these assays produced repellency results similar to the laboratory studies, they did not significantly alter the overall conclusions of this review.

The excito-repellency chamber stood out as the preferred methodology, used in over 53% of the studies. This is likely owing to it being one of the earliest standardised techniques [49] for repellency assessment without the use of attractants, and its capacity to separately evaluate contact and spatial repellency. The popularisation of this methodology reflects its adaptability and relative simplicity. Secondly, the hand-in-cage technique was used in 12 studies. As the oldest technique and the method of choice in WHO [18] and EPA [19] protocols, it is widely adopted owing to its ability to simulate mosquito-human interactions. However, it is a methodology more prone to variability, especially as it depends on individual mosquito behaviour in response to an attractant [50]. The human factor in hand-in-cage assays is critical in evaluating results, as odours generated by hormonal variations, sweat, perfumes and lotions may mask the volatile substances of insecticides, affecting insect behaviour. Indeed, the study by De Obaldia et al. [48] suggests that variations in carboxylic acid levels in human skin affect *A. aegypti* attraction, explaining why some individuals are more frequently bitten than others. Such findings underscore the importance of volunteer selection and pre-assay care in repellency studies using attractants. WHO and EPA protocols for hand-in-cage assays provide general guidance on volunteer selection and pre-assay care, such as using multiple volunteers, random volunteer selection and skin cleaning. However, none of the 18 studies that used humans as attractants provided information about the selected volunteers (most used the same volunteer across multiple assays), and only five studies [12, 29, 31, 34, 51] detailed pre-assay care, such as washing the treated skin with water, soap and ethanol. The fact that none of the studies using human attractants followed WHO or EPA protocol recommendations suggests an inherent difficulty (in volunteer selection) in hand-in-cage assays and explains why most studies avoid using attractants in repellency tests.

These observations lead to the third most used method in the studies: the HITSS system, which has gained relevance as it allows for rapid evaluation of multiple insecticide effects, including contact and spatial repellency, in a standardised format, with a small mosquito sample size (fewer than 30) and ease of execution and interpretation. This versatility and ease of standardisation are reflected in the fact that, although used in fewer studies than hand-in-cage (only 8), HITSS accounts for over 25% of the assays, while hand-in-cage represents ~10% of the repellency assays reviewed.

Mosquito age and physiological status (e.g. gravid females, nutritional status, fat body proportion, etc.) are crucial in repellency assays, as they influence the responsiveness of odour receptors and the motivation for host-seeking [23, 24, 52–54]. All available protocols recommend using nulliparous females, up to 10 days post-emergence, fed only on sugar and subjected to 24-h fasting before testing. The vast majority of studies followed the recommendation of using young, fasted females, but five studies were unclear regarding the age and physiological status of the females used [26, 35, 37–39]. Two studies [40, 41] stood out for using males, gravid females and blood-fed females in repellency assays, aiming to determine whether these insects respond similarly to fasted females. Although repellent effects for these insect groups were observed in some experiments, both manuscripts showed great variability in results and highlighted the need for further studies on repellency behaviour in these insect groups. According to Said [40], understanding repellent effects in males is important because, in addition to being part of the ecosystem and potentially subject to resistance selection, the presence of males affects female host-seeking behaviour [54].

One of the issues raised after analysing the repellency studies was the lack of clarity on how tested doses were defined. As available protocols, particularly those from WHO and EPA, are focused on testing new compounds with repellent effects, there are no guidelines for defining doses to be tested, although the importance of testing serial doses to identify effective repellent doses is emphasised. However, the studies compiled in this review sought to understand the repellent effect of insecticides commonly used for vector control or for insecticide resistance assays in laboratory settings. It would thus be expected that the insecticide doses used in repellency assays be determined based on lethal dose (LD). Indeed, most studies that identified insects as resistant to insecticides reported the tested doses and, consequently, the LDs used to determine resistance or susceptibility. However, only four studies [30, 45–47] used LD data to define the insecticide dose tested for repellency. None of the other 42 studies mentioned the rationale for the doses used in their repellency assays.

Another consideration when testing the repellent effect of insecticides is the choice of compound, whether to use technical grade or formulated products. While technical grade insecticides are tested only with the diluent (generally acetone or an oil-based solvent), formulated insecticides may contain more than one active ingredient and several other compounds that alter volatility and surface fixation, increasing the complexity of result interpretation and making it difficult to determine whether the repellent effect is due to the main insecticide, another ingredient, or the combination of compounds [55]. The ease of interpreting results with isolated (technical grade) insecticides is reflected in the fact that only seven studies tested formulated insecticides for repellency [26, 35, 39, 42–44, 56]. However, formulated insecticides are commonly used in society owing to their availability in pharmacies and supermarkets, and the impact of their use on both resistance selection and repellent effect remains underexplored [42, 56].

Regarding the repellent effect of insecticides, despite the predominance of assays using DDT, deltamethrin and permethrin (reflecting the historical and current importance of these compounds in vector control programmes and insecticide resistance testing), virtually all insecticides showed a repellent effect, generally with a higher frequency of contact repellency than spatial repellency. Although repellency is common among insecticides, variation in repellent effects across different insecticides was observed, even within the same class, such as pyrethroids or organochlorines. This variance reflects subtle differences in chemical structure and pharmacokinetic/pharmacodynamic interactions. While pyrethroids and organochlorines predominantly act on voltage-gated sodium channels (VGSC) or GABA receptors in the nervous system, small molecular differences between compounds can alter volatility, cuticular penetration rate, receptor binding affinity, susceptibility to detoxifying enzymes and the ability to induce sublethal irritation [57]. For instance, the persistence and volatility of a pyrethroid such as deltamethrin may differ from those of permethrin, leading to distinct spatial repellency profiles over time. Moreover, insecticide formulation and the application substrate also influence bioavailability and, consequently, repellent capacity [58].

Insecticide resistance represents a growing challenge for vector control. Although resistance research has traditionally focused on mortality and knockdown, the impact of resistance on mosquito avoidance behaviour, such as spatial and contact repellency, has received limited attention. The aggregated repellency data from the selected studies suggest that insecticide-resistant *A. aegypti* exhibit reduced evasive behaviour compared with susceptible counterparts. This observation supports the idea that selection for insecticide resistance may co-select for or negatively influence the sensory and behavioural mechanisms of vector evasion [59–63]. Unfortunately, the methodological variation in the selected studies does not allow for a meta-analysis to test the hypothesis statistically. While this review primarily focuses on the direct repellent effects of insecticides on mosquitoes, it is important to note that these effects also have significant implications for insecticide-treated surfaces such as insecticide-treated nets (ITNs) and indoor residual spraying (IRS). Repellency can lead to mosquitoes avoiding contact with these surfaces, thereby reducing their exposure to the insecticide’s lethal effects. This behavioural avoidance, often termed ‘exophily’ or ‘exophagy’, can impact the overall effectiveness of these control tools, even in the absence of physiological resistance [64, 82].

The molecular and physiological basis for reduced repellency in resistant insects can be multifactorial. Resistance mechanisms, such as increased activity of metabolic enzymes (e.g. cytochrome P450s, glutathione S-transferases and carboxylesterases), can not only internally detoxify the insecticide but also modulate chemoreceptor sensitivity or metabolise volatile compounds that might act as warning signals [31–34]. Enhanced detoxification capacity may allow the insect to tolerate sublethal doses of insecticide in its system or cuticle for longer, delaying or diminishing the perception of aversive stimuli and consequently reducing repellency response [62]. Additionally, upregulated expression of genes involved in metabolic resistance may impose an energetic cost, potentially affecting sensory and behavioural systems’ performance and making the insect less responsive to repellent stimuli. Another relevant resistance mechanism involves target-site mutations, such as knockdown resistance (*kdr*) mutations in VGSC for pyrethroids, or mutations in GABA receptors (GABAR) for organochlorines [65–68]. While the primary effect is reduced mortality, decreased binding affinity at the target site may have subtle implications for perception. Suppose the insecticide cannot effectively activate sensory neurons or the central nervous system to generate an aversive stimulus (e.g. irritation, disorientation), even at sublethal doses or upon initial contact. In that case, the evasive response may be attenuated or delayed [12, 60, 63]. Target-site insensitivity means that, although the insect may be exposed to the chemical, it does not ‘feel’ the danger sufficiently to trigger a strong behavioural escape response. Cuticular resistance, characterised by cuticle thickening or compositional changes, also contributes to reduced repellency by decreasing insecticide penetration rate. Slower or reduced absorption may mean that the insecticide concentration at sensory receptor sites or action targets does not reach the threshold necessary to induce repellency as rapidly or intensely in resistant insects [31, 34].

In this context, the studies by Andreazza and coworkers [31–34] stood out for attempting to understand the role of odour receptors in repellency behaviour. These works observed that knockout populations for odour receptor genes were repelled in 100% of experiments, a result that contradicts the hypothesis that loss of olfactory receptor function would lead to loss of volatile compound perception. Several possible explanations exist for this finding. First, there is likely functional redundancy in the mosquito olfactory detection systems, meaning the absence of one gene may be compensated by others in the same functional family [31, 32]. Moreover, other sensory pathways (such as ionotropic receptors or trigeminal detection mechanisms) may be involved in the repellency response, especially to deltamethrin and DDT, which have irritant effects (detected via tarsal contact) in addition to olfactory ones [8, 64, 66]. Another possibility is that the knockout affected only specific odour receptors but not the general receptors involved in detecting the compounds used in the tests. These studies open space for broader discussions on the complexity of repellent behaviour and the multiple sensory systems involved, particularly in light of the diversity of compounds used and the genetic variability of the populations studied.

## Conclusions

This systematic review highlights the complex and context-dependent nature of *A. aegypti* repellency to insecticides. Our findings confirm that both contact and spatial repellency are observed across various insecticide classes, although pyrethroids show the most consistent effect. However, methodological variability makes it difficult to compare among studies and draw more accurate conclusions through meta-analyses. Not only that, but we also found that many studies do not comply with some aspects of the WHO and EPA guidelines, especially when using humans as attractants. These findings underscore the need for standardised methodologies in future research and offer a new perspective on the efficacy of insecticide-based vector control strategies. In summary, this study provides a crucial synthesis of the literature, revealing that repellent behaviour remains an important, yet often overlooked, defence mechanism in *A. aegypti*, suggesting that the non-lethal effects of repellency may contribute to the lack of effectiveness of insecticidal tools and the increase of insecticide-resistant populations.

## Supplementary Information

Additional file 1. PRISMA checklist

Additional file 2. Dataset S1. Excel table of extracted data of selected manuscripts

## Data Availability

All data are available in the main text and additional files.

## Abbreviations

**EPA**: Environmental protection agency
**ERC**: Excito-repellency chamber
**HITSS**: High throughput screening system
***kdr***: Knockdown resistance
LD: Lethal dose
**VGSC**: Voltage gate sodium channel
