# Supplementary material for: Repellent effects of insecticides against Aedes aegypti: a systematic review
Source: Parasit Vectors. 2025 Dec 16;18:504. doi: 10.1186/s13071-025-07140-z (PMC12709794; doi:10.1186/s13071-025-07140-z)
Supplement: Supplementary file 1 — Additional file 1. PRISMA checklist [file 13071_2025_7140_MOESM1_ESM.docx]

**Supporting Material**

**Repellency of *Aedes aegypti* to Insecticides: A Systematic Review**

Leonardo Barbosa Koerich^1,2†*^; Artur Metzker Serravite^1†^; Pedro Henryque de Castro^1^; Julia Paula Rabelo^1^; Pedro Horta Andrade^1^, Daniel Milagre Marques^1^; de; Marcos Horácio Pereira^3^; Mauricio Vianna Sant’Anna^3^; Nelder Figueiredo Gontijo^3^; Juliana Maria Trindade Bezerra^4^; Grasielle Caldas D’Ávila Pessoa^1*^

**PRISMA 2020 Checklist: Main Systematic Review**

| **Section/Topic** | **Item Number** | **Checklist Item** | **Reported on Page/Section in Manuscript** |
| --- | --- | --- | --- |
| **Title** | 1 | Identify the report as a systematic review. | The title clearly states "A Systematic Review." |
| **Abstract** | 2 | Provide a structured summary. | The manuscript includes a structured abstract with background, methods, results, and conclusions. |
| **Introduction** | 3 | Describe the rationale. | The Introduction section explains the need for a systematic review on repellency of *Aedes aegypti* to insecticides. |
|  | 4 | State the objectives. | The final paragraph of the Introduction clearly states the study objectives. |
| **Methods** | 5 | Describe the eligibility criteria. | The section "Study Selection and Data Extraction" outlines the inclusion and exclusion criteria. |
|  | 6 | Describe the information sources. | The section "Literature Search Strategy" lists the databases searched. |
|  | 7 | Describe the search strategy. | The section "Literature Search Strategy" describes the search terms and methodology. |
|  | 8 | Describe the selection process. | The "Study Selection and Data Extraction" section details the screening and selection of articles. |
|  | 9 | Describe the data collection process. | The section "Study Selection and Data Extraction" explains how data were extracted from the included articles. |
|  | 10 | List and define all outcomes. | This information is spread across the "Results" section, describing the variables and outcomes assessed (e.g., contact vs. spatial repellency, insecticide types, etc.). |
|  | 11 | Describe any methods used to assess risk of bias in individual studies. | Not explicitly stated in the manuscript. This may need to be added to the Methods section. |
|  | 12 | Describe any methods used to synthesize results. | The "Statistical Analysis" section describes the statistical tests (e.g., Pearson’s chi-square test) used to synthesize the data. |
|  | 13 | Describe any methods used to investigate publication bias. | Not explicitly stated in the manuscript. This may need to be added to the Methods section. |
| **Results** | 14 | State the number of studies and participants. | **Figure 1** provides a flowchart showing the number of studies at each stage of the review. The final number of included studies is reported in the Results. |
|  | 15 | Summarise the characteristics of the included studies. | The "Results" section provides descriptive information about the included studies (e.g., publication year, compounds evaluated). |
|  | 16 | Present results of risk of bias assessment. | Not applicable, as this was not mentioned in the Methods section. |
|  | 17 | Present results of the synthesis. | The "Results" section, including figures and tables, presents the synthesized findings on repellency. |
|  | 18 | Present results of any additional analyses. | The "Statistical Analysis" section and figures present the results of specific analyses (e.g., chi-square test). |
| **Discussion** | 19 | Discuss the limitations of the body of evidence. | The "Discussion" section addresses limitations of the included studies, such as the variability of methodologies. |
|  | 20 | Provide a general interpretation of the results. | The "Discussion" section interprets the overall findings and their implications. |
| **Other Information** | 21 | Describe any funding sources. | The manuscript includes a section acknowledging funding sources. |
|  | 22 | Describe any registered protocol. | Not applicable, as the protocol was not registered. |
|  | 23 | Describe any competing interests. | The manuscript does not explicitly state competing interests. This may need to be added. |

**String used for search in databases**

(Aedes aegypti[all]) AND (repellency[all]) OR (insect repellents[all])) AND (((organochlorines[all]) OR (pyrethroids[all]) OR (organophosphates[all]) OR (carbamates[all]) OR (neonicotinoids[all]) OR (insect growth regulators[all])) OR ((repellency[all]) AND (organochlorines[all])) OR ((repellency[all]) AND (pyrethroids[all])) OR ((repellency[all]) AND (organophosphates[all])) OR ((repellency[all]) AND (carbamates[all])) OR ((repellency[all]) AND (neonicotinoids[all])) OR ((repellency[all]) AND (insect growth regulators[all])) OR ((insect repellents[all]) AND (organochlorines[all])) OR ((insect repellents[all]) AND (pyrethroids[all])) OR ((insect repellents[all]) AND (organophosphates[all])) OR ((insect repellents[all]) AND (carbamates[all])) OR ((insect repellents[all]) AND (neonicotinoids[all])) OR ((insect repellents[all]) AND (insect growth regulators[all])))
